# Nomenclatural review of *Acalypha* (Euphorbiaceae) of the Western Indian Ocean Region (Madagascar, the Comoros Archipelago, the Mascarene Islands and the Seychelles Archipelago)

**DOI:** 10.3897/phytokeys.108.27284

**Published:** 2018-09-10

**Authors:** Iris Montero Muñoz, José María Cardiel, Geoffrey A. Levin

**Affiliations:** 1 Departamento de Biología, Facultad de Ciencias, Universidad Autónoma de Madrid, Ciudad Universitaria de Cantoblanco, 28049, Madrid, Spain Universidad Autónoma de Madrid Madrid Spain; 2 Illinois Natural History Survey, Prairie Research Institute, University of Illinois, 1816 S Oak St., Champaign, IL 61820, USA University of Illinois Champaign United States of America; 3 currrent address: Canadian Museum of Nature, P.O. Box 3443, Station D, Ottawa, ON K1P 6P4, Canada Canadian Museum of Nature Ottawa Canada

**Keywords:** *
Acalypha
*, Comoros, Euphorbiaceae, Madagascar, Mascarenes, nomenclature, Seychelles, synonymy

## Abstract

This work presents a critical nomenclatural review of the *Acalypha* species of the Western Indian Ocean Region (Madagascar, the Comoros Archipelago, the Mascarene Islands and the Seychelles Archipelago). This is the first treatment of *Acalypha* of Madagascar since Leandri’s monograph in 1942. A total of 151 scientific names related to *Acalypha* from this region are treated. We recognise 35 species (28 native and seven introduced), treat 93 names as synonyms (28 of them for the first time) and identify three as doubtful or excluded names. We designate lectotypes for 41 names, make two new combinations and propose one new name.

## Introduction

This work is part of the taxonomic and biogeographic revision of *Acalypha* (Euphorbiaceae) in the Western Indian Ocean Region (WIOR). According to the Taxonomic Databases Working Group standards (Brummit 2001), this region includes Madagascar, the Mascarene Islands (Mauritius and the French Department of Réunion), the Comoros Islands (Union of the Comoros and the French Department of Mayotte) and the Seychelles Archipelago. There are no species of *Acalypha* known from any of the smaller Scattered Islands of the Western Indian Ocean.

The first two species of Madagascan *Acalypha* (*A.filiformis* and *A.venosa*) were described by Poiret (1804) in Lamarck’s encyclopaedia. More than half a century later, Baillon (1861) published *Euphorbiacées Africaines, Afrique Orientale* (*Bourbon, Maurice, Madagascar, Zanguebar* etc.), where 21 species of *Acalypha* are cited, of which 19 are from the study area. Baker (1883, 1884), 20 years later, described four new species. In 1891, Baillon, in his contribution to Grandidier`s *Histoire Physique, Naturelle et Politique de Madagascar*, provided 10 excellent plates of *Acalypha* species from Madagascar, five of which are cited for the first time (Baillon 1891). These plates are not accompanied by descriptions or any additional information. Soon thereafter, Baillon (1892, 1895a, 1895b) published in several chapters his *Liste des Plantes de Madagascar* citing 32 *Acalypha* species, six of which were newly described.

In the treatment of *Acalypha* for Engler´s Das Pfanzenreich, Pax and Hoffmann (1924) included 31 species from WIOR. In this work, *Acalypha* was formally divided into three subgenera, “*Euacalypha*”, *Androcephala* and *Linostachys*; the first two occur in WIOR. The most recent work treating Madagascan *Acalypha* (Leandri 1942) is now almost 80 years old. Leandri’s work included complete information about 22 species and 14 varieties of *Acalypha*, of which eight species and six varieties were described for the first time.

Only three floristic works included *Acalypha* species from the other islands of the Western Indian Ocean region. Baker (1877) cited five species from Mauritius and the Seychelles and de Cordemoy (1895) treated five species from Réunion. Most recently, Coode (1982) included five species, three subspecies and seven varieties from the Mascarene Islands.

While undertaking this nomenclatural review, we found 144 names related to *Acalypha* from the study area, many of which involved serious problems of taxonomic identity. All of these names have been evaluated in this work and our interpretation of their current taxonomic status and distribution is presented in Tables 1 and 2. Our aim is to lay the foundation for a complete taxonomic and biogeographical revision of *Acalypha* in the WIOR, a region of particular relevance to the origin and evolution of this genus. Preliminary results of *Acalypha* molecular phylogeny (Levin et al. 2005) suggest that the genus first appeared in Africa, where the highest morphological diversity within the genus is found. We share the same purpose as the recently published WIOR nomenclator of *Croton* (Berry et al. 2017), the second largest genus of the Euphorbiaceae, preceded by *Euphorbia* (Horn et al. 2012) and followed by *Acalypha*.

## Materials and methods

We conducted a thorough bibliographical review and consulted the following herbaria for the Western Indian Ocean *Acalypha* types: B, BM, BREM, BRNU, C, CAS, G, GB, GDC, GH, JE, K, M, MA, MAU, MO, MPU, NY, P, S, US, TAN, TUB and W (acronyms according to Thiers 2018). Scanned images of types from these and other herbaria, available on JSTOR Global Plants (http://plants.jstor.org/), were also consulted.

The accepted species are cited in alphabetical order and all known synonyms are included, except for the introduced species. We include the notation “**syn. nov.**” whenever we newly treat a taxon as a synonym. We provide details of the type collection(s) for each name. Lectotypes are designated after a careful review of the relevant original literature and examination of the nomenclatural types.

Five of the *Acalypha* treated names were illustrated first, without description, in Baillon`s *Histoire Naturelle des Plantes* (Baillon 1891). These are *A.diminuta*, *A.humblotiana*, *A.leptomyura*, *A.madreporica* and *A.polynema*. Except *A.humblotiana*, we consider each of the plates provided, which include extensive vegetative and reproductive morphological details, as the holotypes of the associated names, according to art. 38.8 of the ICN (McNeill et al. 2012).

When two or more syntypes were cited in the protologue, a lectotype is designated, using the best preserved specimen or the one most consistent with the protologue. The remaining syntypes are also cited.

The type locality information is taken mainly from the type specimen labels. We add additional information such as the province name of Madagascar or a modern or accepted spelling of a place name. If the locality is ambiguous or imprecise, we keep the literal citation (in quotation marks) as it appears in the protologue. Barcode numbers of type specimens are cited when available.

Under the “Distribution” section, we list the general distribution of each species in Africa (following Brummit 2001), if they occur there and in the WIOR region. We include the Madagascar provinces or island names within the studied archipelagos where they occur. This information should be taken as preliminary until a more complete study of the genus, in progress, is completed.

In the references section, we only include references which cite *Acalypha* in the WIOR region.

All information gathered as part of this work, including the complete listing of studied specimens, is available online at the regularly updated *Acalypha Taxonomic Information System* website, www.acalypha.es (Cardiel et al. 2018).

## Results and discussion

In the present work, we record 151 published scientific names related to *Acalypha* of the WIOR (Table 1). Of these, 35 are accepted names, 93 are considered synonyms and three are doubtful or excluded (*A.neptunica* Müll.Arg., *A.spiciflora* Burm.f. and *A.venosa* Poir.). We also include 19 *nomina nuda* found in literature and one *nomen invalidum*. Type specimens are indicated for all valid names and we have designated 41 lectotypes.

**Table 1.** Taxa included in *Acalypha* of the Western Indian Ocean Region. Accepted names in bold.

Published names	Accepted names in this paper
*A.acuminata* Vahl ex Baill. *nom. illeg*.	***A.burmanii* I.Montero & Cardiel nom. nov.**
*A.aldabrica* Pax & K.Hoffm.	***A.claoxyloides* Hutch.**
A.amentaceaRoxb.subsp.wilkesiana (Müll.Arg.) Fosberg	***A.wilkesiana Müll.Arg.***
*A.andringitrensis* Leandri	***A.radula* Baker**
*A.arborea* Comm. in Poir. *nom. nud.*	***A.filiformis* Poir.**
*A.aspretorum* Leandri *nom. nud.*	***A.medibracteata* Radcl.-Sm. & Govaerts**
***A.bailloniana*** Müll.Arg.	
*A.bakeriana* Baill.	***A.emirnensis* Baill.**
*A.baronii* Baker	***A.emirnensis* Baill.**
***A.boinensis* Leandri**	
*A.buchenavii* Müll.Arg.	***A.spachiana* Baill.**
***A.burmanii* I.Montero & Cardiel** *nom. nov.*	
***A.chibomboa* Baill.**	
***A.claoxyloides* Hutch.**	
*A.cloiselana* M. Denis in Leandri *nom. nud.*	***A.gracilipes* Baill.**
*A.codonocalyx* Baill.	***A.chibomboa* Baill.**
*A.colorata* (Poir.) Spreng.	***A.integrifolia* Willd.**
*A.commersoniana* Baill. *nom. nud.*	***A.integrifolia* Willd.**
*A.commersoniana* Baill. ex Müll.Arg.	***A.integrifolia* Willd.**
A.commersonianavar.acutifolia Müll.Arg.	***A.integrifolia* Willd.**
A.commersonianavar.acutifoliaf.concolor Müll.Arg.	***A.integrifolia* Willd.**
A.commersonianavar.acutifoliaf.purpurea Müll.Arg.	***A.integrifolia* Willd.**
A.commersonianavar.acutifoliaf.purpureo-marginata Müll.Arg.	***A.integrifolia* Willd.**
A.commersonianavar.brevifolia Baill. ex Müll.Arg.	***A.integrifolia* Willd.**
A.commersonianavar.concolor Baill. *nom. nud.*	***A.integrifolia* Willd.**
A.commersonianavar.discolor Baill. *nom. nud.*	***A.integrifolia* Willd.**
A.commersonianavar.gracilipes (Baill.) Müll.Arg.	***A.gracilipes* Baill.**
A.commersonianavar.longifolia Müll.Arg.	***A.integrifolia* Willd.**
A.commersonianavar.obtusifolia Müll.Arg.	***A.integrifolia* Willd.**
A.commersonianavar.obtusifoliaf.colorata (Poir.) Müll.Arg.	***A.integrifolia* Willd.**
A.commersonianavar.obtusifoliaf.discolor Müll.Arg.	***A.integrifolia* Willd.**
A.commersonianavar.obtusifoliaf.unicolor Müll.Arg.	***A.integrifolia* Willd.**
A.commersonianavar.parvifolia Baill. *nom. nud.*	***A.integrifolia* Willd.**
A.commersonianavar.parvifolia Baill. ex Müll.Arg.	***A.integrifolia* Willd.**
*A.commersonii* Baill. *nom. nud.*	***A.gracilipes* Baill.**
*A.comorensis* Pax	***A.chibomboa* Baill.**
A.crenatavar.glandulosa Müll.Arg.	**A.lanceolatavar.glandulosa (Müll.Arg.) Radcl.-Sm.**
***A.decaryana* Leandri**	
***A.diminuta* Baill.**	
*A.discolor* Bojer *nom. nud.*	***A.integrifolia* Willd.**
***A.emirnensis* Baill.**	
A.emirnensisvar.bara Leandri	***A.emirnensis* Baill.**
A.emirnensisvar.jabohaziana Leandri	***A.emirnensis* Baill.**
***A.fasciculata* Müll.Arg.**	
A.fasciculatavar.humbertiana Leandri	***A.fasciculata* Müll.Arg.**
A.fasciculatavar.lyallii (Baker) Leandri	***A.fasciculata* Müll.Arg.**
***A.filiformis* Poir.**	
A.filiformisvar.arborea Poir.	***A.filiformis* Poir.**
A.filiformisvar.goudotiana (Baill.) Govaerts	***A.urophylla* Boivin ex Baill.**
A.filiformisvar.ovalifolia (Baill.) Govaerts	***A.ovalifolia* Baill.**
A.filiformisvar.pervilleana (Baill.) Govaerts	***A.paxii* Aug.DC.**
A.filiformisvar.urophylla (Boivin ex Baill.) Govaerts	***A.urophylla* Boivin ex Baill.**
A.filiformisvar.urophylloides (Pax & K.Hoffm.) Govaerts	***A.paxii* Aug.DC.**
***A.fimbriata* Schumach. & Thonn.**	
*A.fryeri* Hutch.	***A.claoxyloides* Hutch.**
*A.gagnepainii* Leandri *nom. illeg.*	***A.medibracteata* Radcl.-Sm. & Govaerts**
A.gagnepainiivar.calcicola Leandri	***A.medibracteata* Radcl.-Sm. & Govaerts**
*A.goudotiana* Baill.	***A.urophylla* Boivin ex Baill.**
***A.gracilipes* Baill.**	
*A.hildebrandtii* Baill.	***A.radula* Baker**
***A.hispida* Burm.f.**	
*A.hologyna* Baker	***A.emirnensis* Baill.**
***A.humbertii* Leandri**	
*A.humblotiana* Baill.	***A.paxii* Aug.DC.**
***A.indica* L.**	
*A.indicavar.bailloniana* (Müll.Arg.) Hutch.	***A.bailloniana* Müll.Arg.**
***A.integrifolia* Willd.**	
A.integrifoliasubsp.marginata (Poir.) Coode	***A.marginata* (Poir.) Spreng.**
A.integrifoliasubsp.marginatavar.crateriana Coode	***A.marginata* (Poir.) Spreng.**
A.integrifoliasubsp.marginatavar.saltuum Coode	***A.marginata* (Poir.) Spreng.**
A.integrifoliasubsp.panduriformis Coode	***A.marginata* (Poir.) Spreng.**
*A.integrifoliavar.colorata* (Poir.) Pax & K.Hoffm.	***A.integrifolia Willd.***
A.integrifoliavar.concolor (Müll.Arg.) Pax & K.Hoffm.	***A.integrifolia* Willd.**
A.integrifoliavar.gracilipes (Baill.) Pax & K.Hoffm.	***A.gracilipes* Baill.**
A.integrifoliavar.longifolia (Müll.Arg.) Coode	***A.integrifolia* Willd.**
A.integrifoliavar.parvifolia (Baill. ex Müll.Arg.) Pax & K.Hoffm.	***A.integrifolia* Willd.**
*A.juliflora* Pax	***A.rottleroides* Baill.**
***A.lamiana* (Leandri) I.Montero & Cardiel** *comb. nov.*	
**A.lanceolatavar.glandulosa (Müll.Arg.) Radcl.-Sm.**	
*A.lantanaefolia* Bojer *nom. nud.*	***A.filiformis* Poir.**
***A.leonii* Baill.**	
A.leoniivar.perrierana Leandri	***A.leonii* Baill.**
***A.lepidopagensis* Leandri**	
***A.leptomyura* Baill.**	
***A.linearifolia* Leandri**	
*A.lyallii* Baker	***A.fasciculata* Müll.Arg.**
*A.madagascariensis* Pax & K.Hoffm.	***A.fasciculata* Müll.Arg.**
*A.madreporica* Baill.	***A.fasciculata* Müll.Arg.**
***A.marginata* (Poir.) Spreng.**	
***A.medibracteata* Radcl.-Sm. & Govaerts**	
A.medibracteatavar.calcicola (Leandri) Radcl.-Sm. & Govaerts	***A.medibracteata* Radcl.-Sm. & Govaerts**
*A.meiodonta* Baill.	***A.paxii* Aug.DC.**
*A.menabeana* Leandri *nom. nud.*	***A.medibracteata* Radcl.-Sm. & Govaerts**
***A.menavody* (Leandri) I.Montero & Cardiel** *comb. nov.*	
***A.neptunica* Müll.Arg.**	
*A.ovalifolia* Baill.	***A.richardiana* Baill.**
***A.paxii* Aug.DC.**	
***A.perrieri* Leandri**	
*A.pervilleana* Baill.	***A.paxii* Aug.DC.**
***A.poiretii* Spreng.**	
*A.polynema* Baill.	***A.rottleroides* Baill.**
*A.radula* Baker	***A.radula Baker***
*A.reticulata* (Poir.) Müll.Arg.	***A.filiformis* Poir.**
A.reticulatavar.arborea (Poir.) Müll.Arg.	***A.filiformis* Poir.**
A.reticulatavar.cloiselana Leandri	***A.gracilipes* Baill.**
A.reticulatavar.goudotiana (Baill.) Müll.Arg.	***A.urophylla* Boivin ex Baill.**
A.reticulatavar.longifolia Müll.Arg.	***A.integrifolia* Willd.**
A.reticulatavar.longifoliaf.aberrans Müll.Arg.	***A.integrifolia* Willd.**
A.reticulatavar.meiodonta (Baill.) Leandri	***A.paxii* Aug.DC.**
A.reticulatavar.meiodontaf.andronea Leandri	***A.paxii* Aug.DC.**
A.reticulatavar.ovalifolia (Baill.) Müll.Arg.	***A.ovalifolia* Baill.**
A.reticulatavar.pervilleana (Baill.) Müll.Arg.	***A.paxii* Aug.DC.**
A.reticulatavar.urophylla (Boivin ex Baill.) Müll.Arg.	***A.urophylla* Boivin ex Baill.**
A.reticulatavar.urophyllaf.glabrescens Leandri	***A.urophylla* Boivin ex Baill.**
A.reticulatavar.urophyllaf.humblotiana (Baill.) Leandri	***A.paxii* Aug.DC.**
A.reticulatavar.urophyllaf.lamiana Leandri	***A.lamiana* (Leandri) I.Montero & Cardiel comb. nov.**
A.reticulatavar.urophyllaf.longa Leandri	***A.urophylla* Boivin ex Baill.**
A.reticulatavar.urophyllaf.meeusei Leandri	***A.urophylla* Boivin ex Baill.**
A.reticulatavar.urophyllaf.typique Leandri *nom. inval.*	***A.urophylla* Boivin ex Baill.**
A.reticulatavar.urophyllaf.vohitrae Leandri	***A.urophylla* Boivin ex Baill.**
A.reticulatavar.urophylloides Pax & K.Hoffm.	***A.paxii* Aug.DC.**
***A.richardiana* Baill.**	
***A.rottleroides* Baill.**	
*A.salviifolia* Baill. *nom. nud.*	***A.radula* Baker**
***A.spachiana* Baill.**	
A.spachianavar.acutifolia Baill.	***A.spachiana* Baill.**
A.spachianavar.latifolia Baill.	***A.spachiana* Baill.**
A.spachianavar.minor Baill.	***A.spachiana* Baill.**
*A.spiciflora* Burm.f.	***Claoxylonspiciflorum* (Burm.f.) A.Juss.**
A.spicifloravar.menavody Leandri	***A.menavody* (Leandri) I.Montero & Cardiel comb. nov.**
*A.squarrosa* Pax	***A.spachiana* Baill.**
***A.urophylla* Boivin ex Baill.**	
*A.urophylla* Pax *nom. illeg*.	***A.paxii* Aug.DC.**
*A.venosa* Poir.	***Leptonemavenosum* (Poir.) A.Juss.**
***A.vulneraria* Baill.**	
***A.wilkesiana* Müll.Arg.**	
*Caturussessilis* Pet.Thou. in Baill. *nom. nud.*	***A.integrifolia* Willd.**
*Ricinocarpusbaillonianus* (Müll.Arg) Kuntze	***A.bailloniana* Müll.Arg.**
*Ricinocarpushispidus* (Brum.f.) Kuntze	***A.hispida* Brum.f.**
*Ricinocarpuspoiretii* (Spreng.) Kuntze	***A.poiretii* Spreng.**
*Ricinocarpuswilkesianus* (Müll.Arg.) Fosberg	***A.wilkesiana* Müll.Arg.**
*Tragiacolorata* Poir.	***A.integrifolia* Willd.**
*Tragiafiliformis* Poir.	***A.burmanii* I.Monero & Cardiel nom. nov.**
*Tragiafruticosa* Commers. in Baill. *nom. nud.*	***A.integrifolia* Willd.**
*Tragialobata* Wall. *nom. nud.*	***A.integrifolia* Willd.**
*Tragiamacrophylla* Wall. *nom. nud.*	***A.integrifolia* Willd.**
*Tragiamarginata* Poir.	***A.integrifolia* Willd.**
*Tragiaobtusata* Vahl. in Baill. *nom. nud.*	***A.integrifolia* Willd.**
*Tragiareticulata* Poir.	***A.filiformis* Poir.**
*Tragiasalviifolia* Bojer in Baill. *nom. nud.*	***A.radula* Baker**
*Tragiasaxatilis* Bojer in Pax & K.Hoffm. *nom. nud.*	***A.spachiana* Baill.**

In Madagascar, we accept 28 species, 23 native (of which 20 are endemic) and five introduced (Table 2). Leandri (1942) recognised 22 species of *Acalypha* from Madagascar, of which we accept 19 (including *A.gagnepainii* under its correct name, *A.medibracteata*). We consider *A.andringitrensis* to be a synonym of *A.radula*. Our concept of what Leandri called *A.reticulata* is narrower than his; we consider *A.reticulatas.s.* (under its correct name, *A.filiformis*) to be endemic to the Mascarines and treat the Madagascan material Leandri assigned to this species as four distinct species (*A.gracilipes*, *A.lamiana*, *A.paxii* and *A.urophylla*). We exclude the name *Acalyphaspiciflora* (accepted by Leandri), because it is not an *Acalypha*. We report two species, *A.fimbriata* and *A.lanceolata*, not previously cited for Madagascar. We also treat the name *A.madagascariensis*, previously excluded by Leandri, as a synonym of *A.fasciculata*. We anticipate the description of at least 2 more new species from Madagascar based on the material we now have on hand.

**Table 2.** Synoptic table of the species distribution of *Acalypha* of the Western Indian Ocean Region (E: endemic, N: native, I: introduced).

Species	Madagascar	Comoros	Mascarenes	Seychelles
* A. bailloniana *		I		
* A. boinensis *	E			
* A. burmanii *	E			
* A. chibomboa *	N	N		
* A. claoxyloides *				E
* A. decaryana *	E			
* A. diminuta *	E			
* A. emirnensis *	E			
* A. fasciculata *	E			
* A. filiformis *			E	
* A. fimbriata *	I			
* A. gracilipes *	E			
* A. humbertii *	E			
* A. indica *	I	I	I	I
* A. integrifolia *			E	
* A. lamiana *	E			
A. lanceolata var. glandulosa	I		I	
* A. leonii *	E			
* A. lepidopagensis *	E			
* A. leptomyura *	E			
* A. linearifolia *	E			
* A. marginata *			E	
* A. medibracteata *	E			
* A. menavody *	E			
* A. paxii *	N	N		
* A. perrieri *	E			
* A. radula *	E			
* A. richardiana *		E		
* A. rottleroides *	E			
* A. spachiana *	E			
* A. urophylla *	N	N		
* A. vulneraria *	E			
* A. hispida *	I	I	I	I
* A. poiretii *			I	
* A. wilkesiana *	I			I
**Native**	23	4	3	1
**Endemic**	20	1	3	1
**Introduced**	5	3	4	3
**Total**	28	7	7	4

In the Comoros Archipelago, there are seven species of *Acalypha*, four native (one endemic) and three introduced, one of which, *A.bailloniana*, is first reported here. There are seven species on the Mascarene Islands, three native (all endemic) and four introduced. There are four species on the Seychelles, one endemic and three introduced.

### Nomenclatural synopsis of Malagasy, Comoros, Mascarene and Seychelles *Acalypha*

#### 1. Acalypha bailloniana

Taxon classificationPlantaeMalpighialesEuphorbiaceae

Müll.Arg., Linnaea 34: 44. 1865.

Ricinocarpus baillonianus (Müll.Arg.) Kuntze, Revis. Gen. Pl. 2: 617. 1891.
Acalypha indica var. bailloniana (Müll.Arg.) Hutch., Fl. Trop. Afr. 6(1): 904. 1912.

##### Type.

Tanzania: Zanzibar: s.l., 1848, *L. H. Boivin s.n.* (holotype: P [P04809900]!).

##### Distribution.

East Tropical Africa. Comoros Archipelago (Anjouan).

##### Notes.

*Acalyphabailloniana* was considered as a subspecies of *A.indica* L. by Hutchinson (1913) and this treatment was followed in the subsequent floristic works. Radcliffe-Smith (1987, 1996) treated *A.bailloniana* as a synonym of *A.indica*. We consider that *A.bailloniana* is a distinct species which can be distinguished from *A.indica* by its dentante bracts with a prominent central tooth and with glandular hairs (vs. subentire bracts without prominent central tooth and without glandular hairs, in *A.indica*). *A.bailloniana* is distributed in the east coast of tropical Africa and cited for the WIOR region for the first time, where it is probably introduced.

#### 2. Acalypha boinensis

Taxon classificationPlantaeMalpighialesEuphorbiaceae

Leandri, Notul. Syst. (Paris) 10: 268. 1942.

##### Type.

Madagascar. Prov. Mahajanga: Ambongo et Boïna, bassin moyen du Bemarivo, 200 m, Nov 1919, *H. Perrier de la Bâthie 9823* (lectotype, designated here: P [P00508582]!).

##### Syntypes.

Madagascar. Prov. Mahajanga: Maintirano, s.d., *R. Decary 8216* P [P00508588]!; Madagascar. Prov. Mahajanga: Maromandia (Andranosamontana), s.d., *R. Decary 1045* P [P00508585]!; Madagascar. Prov. Mahajanga: Morovoay, s.d., *H. Humbert & H. Perrier de la Bâthie 2350* P [P00508570!, P00508571!, P00508572!, P00508573!, P00508574!]; Madagascar. Prov. Mahajanga: Ambongo et Boïna, 300 m, s.d., *H. Perrier de la Bâthie 9546* P [P00508576]!, *9551* P [P00508577]!; Madagascar. Prov. Toliara: Forêt de Besomaty, entre le Fiherena et l’Isahaina (Mangoky), 750–800 m, s.d., *H. Humbert 11234* P [P00508575]!; Madagascar: s.l., s.d., *Baron 5393* P [P00508586]!, *5450* [n.v.].

##### Distribution.

Madagascar (Antsiranana, Fianarantsoa, Mahajanga, Toliara)

##### References.

Govaerts et al. (2000: 51).

#### 3. Acalypha burmanii

Taxon classificationPlantaeMalpighialesEuphorbiaceae

I.Montero & Cardiel. nom. nov.

urn:lsid:ipni.org:names:60477001-2

Tragia filiformis Poir., Encycl. 7: 727. 1806. Acalyphaacuminata Vahl ex. Baill. Adansonia 1: 267. 1861 *nom. illeg*., non. A.acuminata Benth. (1854).

##### Type.

Madagascar?: s.l., s.d., *Herb. de Lamarck. s.n.* (holotype: P-LAM [P00367371]!).

##### Distribution.

Madagascar (Antsiranana).

##### References.

Müller Argoviensis (1865: 40) as *A.spiciflora* Burm.f.; Müller Argoviensis (1866: 867) as *A.spiciflora*; Baillon (1891: tab. 188) as *A.acuminata* Vahl. ex Baill.; Baillon (1892: 1004) as *A.acuminata*; Heckel (1903: 198) as *A.acuminata*; Palacký (1907: 24) as *A.acuminata*; Pax and Hoffmann (1924: 137) as *A.spiciflora*; Leandri (1942: 269) as *A.spiciflora*.

##### Notes.

*Acalyphaburmanii* is proposed as a new name for *Tragiafiliformis* Poir. We cannot combine *T.filiformis* under *Acalypha* because it is blocked by *A.filiformis* Poir., nor can we use the illegitimate name *A.acuminata* Baill. See also comments under the excluded species *A.spiciflora*.

#### 4. Acalypha chibomboa

Taxon classificationPlantaeMalpighialesEuphorbiaceae

Baill., Adansonia 1: 269. 1861.

##### Type.

Comoros, Anjouan, 1850, *L. H. Boivin s.n.* (holotype: P [P00196274]!).

*Acalyphacodonocalyx* Baill., Adansonia 1: 271. 1861. Type: Comoros: Mohéli, “Ile Mohilla”, s.d., *M. Richard 286* (lectotype, designated here: P [P00196282]!; isolectotype: P [P00196283]!).

*Acalyphacomorensis* Pax, Bot. Jahrb. Syst. 19(1): 95. 1894. Type: Comoros, Anjouan, “Johanna”, Jun–Aug 1875, *J. M. Hildebrandt 1662* (holotype B or WRSL, presumably destroyed; lectotype, designated here: BREM [BREM0001792]!; isolectotypes: K [K000186524!, K000186525!], L [L0241274]!, P [P00196280]!, W [W0004243]!) Syntype: Comoros, Grande Comore, 1886. *C. W. Schmidt 192* [n.v.].

##### Distribution.

Madagascar (Antsiranana, Antananarivo), Comoros Archipelago (Grande Comore, Anjouan, Mohéli, Mayotte).

##### References.

Baillon (1891: Pl. 192 as *A.codonocalyx*); Palacký (1907: 24); Voeltzkow (1917: 447); Pax and Hoffmann (1924: 165); Leandri (1942: 280); Govaerts et al. (2000: 55).

#### 5. Acalypha claoxyloides

Taxon classificationPlantaeMalpighialesEuphorbiaceae

Hutch., Bull. Misc. Inform. Kew 1918: 205. 1918.

##### Type.

Seychelles, Astove, Cosmoledo and Aldabra, Apr 1907, *H. P. Thomasset 243* (lectotype, designated here: K [K000186504]!).

##### Syntypes.

Seychelles, Aldabra, s.d., *W. L. Abbott s.n.* P [P00887488]!, [P00887489]!; Seychelles, Aldabra, Oct-Dec 1892, *J. Fryer 18* K [K000186505]!.

*Acalyphafryeri* Hutch. Bull. Misc. Inform. Kew 1918: 206. 1918. Type. Seychelles, Aldabra, s.d., *J. Fryer 92* (holotype: K [K000186506]!).

*Acalyphaaldabrica* Pax & K.Hoffm., Pflanzenr. 147,16(Heft 85): 136. 1924. Type. Seychelles, Aldabra, s.d., *W. L. Abbott s.n.* (holotype B?, presumably destroyed; lectotype, designated here: P [P00887488]!; isolectotype: P [P00887489]!, **syn. nov**.).

##### Distribution.

Seychelles Archipelago.

##### References.

Hemsley (1919: 130); Hemsley (1919: 131) *A.fryeri*; Pax and Hoffmann (1924: 136) *A.aldabrica*; Fosberg (1974: 263); Renvoize (1975: 152); Robertson (1989: 199); Govaerts et al. (2000: 56).

##### Notes.

*Acalyphaclaoxyloides* is widespread in the Seychelles archipelago. It is very close to *A.pubiflora* (Klotzsch) Baill., known from south-eastern Africa (Botswana, Malawi, Mozambique, South Africa and Zimbabwe), of which it may be a synonym. More studies of the African material, as well as the Australian material treated as A.pubifloravar.australica Radcl.-Sm. (Radcliffe-Smith 1990, 1996), is needed to unravel the *A.pubiflora* complex.

#### 6. Acalypha decaryana

Taxon classificationPlantaeMalpighialesEuphorbiaceae

Leandri, Notul. Syst. (Paris) 10: 284. 1942.

##### Type.

Madagascar. Prov. Toliara: Ambovombe, 20 Aug 1924, *R. Decary 2985* (holotype: P [P00508553]!; isotype W [W1962-0013399]!).

##### Distribution.

Madagascar (Antananarivo, Antsiranana, Fianarantsoa, Toliara).

##### References.

Govaerts et al. (2000: 59); Seebaluck et al. (2015: 150).

##### Notes.

We consider as holotype of *Acalyphadecaryana*, the only specimen with the word “type” hand-writen by Leandri.

#### 7. Acalypha diminuta

Taxon classificationPlantaeMalpighialesEuphorbiaceae

Baill., Hist. Pl. Madag., Atlas, t. 194. 1891.

##### Type.

Plate 194 in Baillon ibid. loc., holotype.

##### Distribution.

Madagascar (Antananarivo, Mahajanga, Toamasina, Toliara).

##### References.

Baillon (1895b: 1197); Palacký (1907: 25); Pax and Hoffmann (1924: 21); Leandri (1935: 42); Leandri (1942: 253); Govaerts et al. (2000: 60).

##### Notes.

*Acalyphadiminuta* was first illustrated, without description, in Baillon`s *Histoire Naturelle des Plantes* (Baillon 1891). The first description of this species, based only on Baillon`s illustration, appears in Engler`s Pflanzenreich (Pax and Hoffmann 1924). This is the only species included in Acalyphasubgen.Androcephala Pax & K.Hoffm.

#### 8. Acalypha emirnensis

Taxon classificationPlantaeMalpighialesEuphorbiaceae

Baill., Adansonia 1: 270. 1861.

##### Type.

Madagascar. Prov. Antananarivo: Antananarivo “in prov. Emirna, prope Tananarivou”, 1833, *M. Bojer s.n.* (holotype: P [P00536723]!; isotypes GD-C [GDC005713]!, P [P00536725]!).

*Acalyphabaronii* Baker, J. Linn. Soc., Bot. 20: 254. 1883. Type: Madagascar: “Central Madagascar”, 1882, *R. Baron 1725* (holotype: K [K000186523]!; isotype P [P00324467]!).

*Acalyphahologyna* Baker, J. Linn. Soc., Bot. 21: 441. 1885. Type: Madagascar: s.l., s.d., *R. Baron 2889* (holotype: K [K000186526]!; isotype P [P00536724]!).

*Acalyphabakeriana* Baill., Bull. Mens. Soc. Linn. Paris 2. 1180. 1895. Type: Madagascar: “Centr. Madag.” *R. Baron 4425* (holotype: P [P00324466]!).

Acalyphaemirnensisvar.bara Leandri, Notul. Syst. (Paris) 10: 282. 1942. Type: Madagascar. Prov. Toliara: Massif of l’Ivakoany, 1928, *H. Humbert 6986* (lectotype, designated here: P [P00508509]!; isolectotype: P [P00508508]! **syn. nov**.).

Syntypes. Madagascar. Prov. Toliara: bassin supérieur du Mandrare: col et sommet de Marosohy, 1000–1400 m, 14–15 Nov 1928, *H. Humbert 6623* P [P00536758]!; Madagascar. Prov. Toliara: Massif de l’Ivakoany, *H. Humbert 12185* P [P00508506!, P00508507]!; Madagascar. Prov. Toliara: entre l’Andohahela et l’Elakelaka, *H. Humbert 13941* [P00324472].

Acalyphaemirnensisvar.jabohaziana Leandri, Notul. Syst. (Paris) 10: 283. 1942. Type: Madagascar. Prov. Mahajanga: “Boina, Jabohazo, près du mont Tsitondroina” Dec 1900, *H. Perrier de la Bâthie 9793* (holotype: P [P00536722]!. **syn. nov**).

##### Distribution.

Madagascar (Antananarivo, Antsiranana, Fianarantsoa, Mahajanga, Toamasina, Toliara).

##### References.

Müller Argoviensis (1866: 804); Baillon (1892: 1003); Baillon (1895a: 1180) as *A.baronii*; Baillon (1895b: 1196) as *A.hologyna*; Palacký (1907: 24, 25); Pax and Hoffmann (1924: 94, 171); Leandri (1942: 281); Govaerts et al. (2000: 50, 61); Govaerts et al. (2000: 67) sub. *A.hologyna*.

#### 9. Acalypha fasciculata

Taxon classificationPlantaeMalpighialesEuphorbiaceae

Müll.Arg., Linnaea 34: 31. 1865.

##### Type.

Madagascar: s.l., s.d., *L. M. A. Du-Petit Thouars s.n.* (lectotype, designated here: P [P00324476]!; isolectotypes: P [P00324495!, P00508505!]).

*Acalyphalyallii* Baker, J. Linn. Soc., Bot. 20: 255. 1883. Acalyphafasciculatavar.lyallii (Baker) Leandri, Notul. Syst. (Paris) 10: 284. 1942. Type: Madagascar: “Central Madagascar”, s.d., *R. Lyall s.n*. (holotype: K [K000186529]! **syn. nov**.).

*Acalyphamadreporica* Baill., Hist. Pl. Madag., Atlas t. 186 (1891). Type: Plate 186 in Baillon ibid. loc., holotype.

*Acalyphamadagascariensis* Pax & K.Hoffm., Pflanzenr. 147, 16 (Heft 85): 162. 1924. Type: Madagascar. Prov. Toliara: Forêt d’Antsianaka, 19 Jan 1882, *H. Humblot 447* [“*449*”] (holotype B, presumably destroyed; lectotype, designated here: P [P00324501]!, **syn. nov.**).

Acalyphafasciculatavar.humbertiana Leandri, Notul. Syst. (Paris) 10: 284. 1942. Type: Madagascar. Prov. Toliara: haute vallée de Mandrare, 8 Nov 1928, *H. Humbert 6514.* Lectotype, designated here: P [P00508503]!; isolectotypes: P [P00324487]!, US [US00096332]! **syn. nov**.). Syntypes. Madagascar. Prov. Toliara: Bassin de la Manampanihy (Sud-Est), col de Fitana, *H. Humbert 6044* P [P00324486]!]; Madagascar. Prov. Toliara: Massif du Beampingaratra, du col de Bevava au sommet de Bekoho, *H. Humbert 6478* P [P00508504]!.

##### Distribution.

Madagascar (Antsiranana, Fianarantsoa, Mahajanga, Toamasina, Toliara).

##### References.

Müller Argoviensis (1866: 851); Baillon (1895a: 1181) *A.madreporica*. and *A.lyallii*; Palacký (1907: 25); Pax and Hoffmann (1924: 94) *A.madreporica*; Pax and Hoffmann (1924: 171); Leandri (1942: 283); Leandri (1948: 186) *A.lyallii*; Leandri (1952)A.fasciculatavar.humbertiana; Govaerts et al. (2000: 61); Govaerts et al. (2000: 73) *A.lyallii*; Govaerts et al. (2000: 74) *A.madreporica*; Schatz (2001: 142) as A.fasciculatavar.humbertiana; Seebaluck et al. (2015: 152) as *A.lyallii*.

##### Notes.

The correct number of the type specimen of *A.madagascariensis* is *Humblot 447.* In the protologue of this name, it is wrongly transcribed as “*Humblot 449*, which corresponds to a specimen of *Psorospermum* (Clusiaceae).

#### 10. Acalypha filiformis

Taxon classificationPlantaeMalpighialesEuphorbiaceae

Poir., Encycl. 6(1): 205. 1804.

##### Type.

Mauritius, “Île de France”, s.d., *P. Commerson s.n.* (lectotype, designated here: P [P05604464]!; isolectotypes: MPU [MPU014933]!, P [P05604471]!).

Acalyphafiliformisvar.arborea Poir., Encycl. 6(1): 205. 1804. Acalyphareticulatavar.arborea (Poir.) Müll.Arg. Linnaea 34: 32. 1865. Type: Reunion, “Elle croît a l’île Bourbon”, 1774, *P. Commerson s.n.* (holotype: P [P05604473]!; isotype: MPU [MPU014949]!).

*Tragiareticulata* Poir., Encycl. 7: 725. 1806. *Acalyphareticulata* (Poir.) Müll.Arg., Prodr. 15(2): 851. 1866. Type: Reunion: “l`Ile -de-Bourbon”, s.d., *P. Commerson s.n.* (holotype: P-LAM [P00382118]!; isotype P [P05604477]!).

*Acalyphaarborea* Commers. in Poir., Encycl. 6: 205. 1804 *nom. nud*.

*Acalyphalantanaefolia* Bojer, Hortus Maurit. 286. 1837 *nom. nud*.

##### Distribution.

Mascarene Islands (Mauritius, Réunion).

##### References.

Bojer (1837: 286); Baillon (1858: 443) as *Tragiareticulata*; Baillon (1861: 266) as *A.arborea*; Müller Argoviensis (1866: 851) as *A.reticulata*; Baker (1877: 316) as *A.reticulata*; Müller Argoviensis (1882: 26) as *A.reticulata*; Pax (1890: 61) as *A.reticulata*; Pax (1894: 96) as *A.reticulata*; de Cordemoy (1895: 342) as *A.reticulata*; Voeltzkow (1917: 447) as *A.arborea*; Pax and Hoffmann (1924: 102) as *A.reticulata*; Leandri (1942: 258) as *A.reticulata*; Coode (1979: 45) as *A.reticulata*; Coode (1982: 69, 76); Govaerts et al. (2000: 62, 99, 105); Seebaluck et al. (2015: 150).

##### Notes.

*Acalyphareticulata* has been usually considered as the accepted name of this species. Leandri (1942) noticed that *A.filiformis* and *A.reticulata* are conspecific, but he kept *A.reticulata* as the accepted name. Applying the rule of priority, the accepted name must be *A.filiformis* and *A.reticulata* should be placed as a synonym.

#### 11. Acalypha fimbriata

Taxon classificationPlantaeMalpighialesEuphorbiaceae

Schumach. & Thonn., Beskr. Guin. Pl. 409. 1827.

##### Type.

Ghana: s.l., s.d., *P. Thonning s.n.* (holotype: C [C10003279]!; isotypes: C [C10003278!, C10003280]!, S [S14-42539]!).

##### Distribution.

West Tropical Africa, West Central Tropical Africa, East Tropical Africa, Northeast Tropical Africa and Southern Africa. Madagascar (Antananarivo, Fianarantsoa, Toliara).

##### Notes.

This is the first time that this species is cited for the WIOR region, where it is almost certainly introduced.

#### 12. Acalypha gracilipes

Taxon classificationPlantaeMalpighialesEuphorbiaceae

Baill., Adansonia 1: 273. 1861.

Acalypha commersoniana var. gracilipes (Baill.) Müll.Arg., Prodr. 15(2): 850. 1866. Acalyphaintegrifoliavar.gracilipes (Baill.) Pax & K.Hoffm., Pflanzenr. 147,16 (Heft 85): 106. 1924.

##### Type.

Madagascar: s.l., s.d., *P. Commerson s.n.* (holotype: P [P04022747]!).

Acalyphareticulatavar.cloiselana Leandri, Notul. Syst. (Paris) 10: 266. 1942. Type: Madagascar: s.l., s.d., *P. Commerson s.n.* (lectotype, designated here: P [P00513166]!, **syn. nov.**).

Syntypes. Madagascar. Prov. Toamasina: Fénérive, *H. Perrier de la Bâthie 9707* [P00513169]!; Madagascar. Prov. Toliara: Fort Dauphin, *J. Cloisel 156* P [P00513165]!; Madagascar. Prov. Toliara: Forêt de Manantantely, *H. Humbert 5835* P [P00513167]!, P [P00513168]!; Madagascar. Prov. Toliara: Fort Dauphin, *G. F. Scott-Elliot 2493* P [P00513171]!; Madagascar: s.l., s.d., *R. Baron 5980* P [P00513164]!; *R. Baron 6420* [P00324562]!.

*Acalyphacloiselana* Denis ex Leandri, Notul. Syst. (Paris) 10: 266. 1942 *nom. nud.* as synonym of A.reticulatavar.cloiselana Leandri.

*Acalyphacommersonii* Baill. ex Leandri, Notul. Syst. (Paris) 10: 266. 1942 *nom. nud.* as synonym of A.reticulatavar.cloiselana Leandri.

##### Distribution.

Madagascar (Antsiranana, Fianarantsoa, Toamasina, Toliara).

##### References.

Müller Argoviensis (1866: 850) as A.commersonianavar.gracilipes; Baillon (1892: 1004); Palacký (1907: 25); Pax and Hoffmann (1924: 106) as A.integrifoliavar.gracilipes; Govaerts et al. (2000: 69, 100).

##### Notes.

*Acalyphagracilipes* has been usually treated as a variety of *A.integrifolia* Willd., which is endemic of Mascarene Islands, but *A.gracilipes* can be clearly distinguished by its elliptic-lanceolate leaves, denticulate female bracts and glabrous and glandular ovaries, vs. linear-lanceolate leaves, entire female bracts and hispidulous and echinate ovary in *A.integrifolia*.

*A.gracilipes* can be distinguished from both *A.urophylla* Boivin ex Baill. and *A.paxii* Aug.D.C. mainly by its glabrous leaves with crenate to subdentate margins and obtuse to subacute apices, glabrous female bracts and glabrous ovaries with minute sessile glands, vs. pubescent leaves with serrate margins and acuminate (*A.urophylla*) or usually caudate (*A.paxii*) apices, pubescent female bracts and hispidulous ovaries with long papillae. In addition, the female bracts of *A.paxii* have a prominent central tooth, which is absent in both *A.gracilipes* and *A.urophylla*.

Leandri (1942) included the collection *H. Perrier de la Bâthie 9746* as Acalyphareticulatavar.cloiselana Leandri, however the specimen of this collection in P (P00513170) corresponds to *A.urophylla*.

#### 13. Acalypha hispida

Taxon classificationPlantaeMalpighialesEuphorbiaceae

Burm.f., Fl. Ind. 303, pl. 61, f. 1. 1768.

##### Type.

Habitat in India, Tab. 61 in Burm. f., loc. cit. 302. 1768.

##### Distribution.

Introduced in Tropical Africa and the WIOR. Madagascar (Toliara).

##### References.

Bojer (1837: 25); Baillon (1861: 274); Palacký (1907: 25); Robertson (1989: 199).

##### Notes.

This shrub, native to Melanesia or Malesia, is frequent in gardens throughout the tropics and rarely appears naturalised. As all plants are pistillate, it can only reproduce clonally. We found collections from Madagascar and Seychelles where it is cultivated. It has been reported from Madagascar (Palacký 1907), Mauritius (Bojer 1837; Baillon 1861), Réunion (Baillon 1861: 274) and the Seychelles (Robertson 1989).

#### 14. Acalypha humbertii

Taxon classificationPlantaeMalpighialesEuphorbiaceae

Leandri, Notul. Syst. (Paris) 10: 274. 1942.

##### Type.

Madagascar. Prov. Toliara: vallées du Mangoky et de l’Isahaina, aux environs de Beroroha, 200 m, Oct 1933, *H. Humbert 11289* (lectotype, designated here: P [P00508400]!; isolectotype: P [P00508399]!).

##### Syntypes.

Madagascar. Prov. Toliara: Bassin supérieur de Mandrare du Sud-Est, entre le col de Vavara et la vallée de la Manambolo, 700–1200 m, 20–22 Dec. 1928, *H. Humbert 6758* P [P00508401!, P00508402!, P00508403!, P00508404]!).

##### Distribution.

Madagascar (Toliara).

##### References.

Govaerts et al. (2000: 67).

#### 15. Acalypha indica

Taxon classificationPlantaeMalpighialesEuphorbiaceae

L., Sp. Pl. 2: 1003. 1753.

##### Type.

India: s.l., s.d., *Herb. Hermann 3: 2*, #*34*. (lectotype, designated by Radcliffe-Smith (1986: 65): BM; isolectotype: BM).

##### Distribution.

Widely distributed in the Paleotropics and introduced in the Americas. In the WIOR, it is found only in disturbed areas and almost certainly is introduced there. Madagascar (Antananarivo, Antsiranana, Fianarantsoa). Comoros Archipelago (Anjouan, Mohéli, Mayotte). Mascarene Islands (Mauritius, Réunion). Seychelles Archipelago.

##### References.

Bojer (1837: 285); Baillon (1861: 274); Müller Argoviensis (1865: 42; 1866: 868); Baker (1877: 314); Müller Argoviensis (1882: 27); Baillon (1895b: 1197); de Cordemoy (1895: 342); Palacký (1907: 25); Voeltzkow (1917: 447); Hemsley (1919: 148); Pax and Hoffmann (1924: 33); Leandri (1935: 43); Leandri (1942: 256); Renvoize (1975: 152); Coode (1982: 69, 78); Robertson (1989: 200); Govaerts et al. (2000: 68).

#### 16. Acalypha integrifolia

Taxon classificationPlantaeMalpighialesEuphorbiaceae

Willd., Sp. Pl. 4(1): 530. 1805.

##### Type.

Mauritius, s.l., s.d., *Anonymous*, *s.n.* (lectotype designated by Coode (1978: 39): B [B-W17834-020]).

*Tragiacolorata* Poir., Encycl. 7: 725. 1806. *Acalyphacolorata* (Poir.) Spreng., Syst. Veg. 3: 879. 1826. Acalyphacommersonianavar.obtusifoliaf.colorata (Poir). Müll.Arg., Prodr. 15(2): 850. 1866. *Acalyphaintegrifoliavar.colorata* (Poir.) Pax & K.Hoffm. Pflanzenr. 147,16 (Heft 85): 106. 1924. Type: Mauritius: “Cete plante croit dans les indes orientales, & à l’Ile de France,”, s.l. s.d., *P. Commerson s.n*. (holotype: P-LAM [P00382140]!).

Acalyphareticulatavar.longifolia Müll.Arg., Linnaea 34: 32. 1865. Acalyphaintegrifoliavar.longifolia (Müll.Arg.) Coode, Kew Bull. 34: 41. 1979. Type: Mauritius: s.l., s.d., *L. Bouton s.n.* (holotype: GDC [G00324522]!, **syn. nov.**).

Acalyphareticulatavar.longifoliaf.aberrans Müll.Arg., Linnaea 34: 32. 1865. Type: Mauritius: “In sylvis Mauritii”, 1833, *M. Bojer s.n.* (holotype: GDC [G00324521]!).

*Acalyphacommersoniana* Baill. ex Müll.Arg., Prodr. 15(2): 849. 1866. Acalyphacommersonianavar.brevifolia Baill. ex Müll.Arg., Prodr. 15(2): 850. 1866. Type: Mauritius: “Cum var. praecedentibus”, s.d., *Anonymous*, *s.n.* (*Hb. Willd. fol. 17834 pag. 1*) (lectotype, designated here: B [B-W17834-010]!).

Syntypes. Mauritius: s.l., s.d., *L. Bouton s.n.* GDC [G00324543]!.

Acalyphacommersonianavar.acutifolia Müll.Arg., Prodr. 15(2): 849. 1866. Acalyphacommersonianavar.acutifoliaf.purpurea Müll.Arg., Prodr. 15(2): 849. 1866. Type: Mauritius: s.l., s.d., *F. W. Sieber 181 pr. p.* (lectotype, designated here: GDC [G00324550]!). Syntype: Mauritius: s.l., s.d., *L. Bouton s.n.* GDC [G00324559]!.

Acalyphacommersonianavar.acutifoliaf.purpureo-marginata Müll.Arg., Prodr. 15(2): 849. 1866. Type: Mauritius: s.l., s.d., *Hb. Boiss. s.n.* (holotype: GDC [G00324557]!; isolectotype: K [K000431097]!, **syn. nov.**).

Acalyphacommersonianavar.acutifoliaf.concolor Müll.Arg., Prodr. 15(2): 849. 1866. Acalyphaintegrifoliavar.concolor (Müll.Arg.) Pax & K.Hoffm, Pflanzenr. 147,16 (Heft 85): 106. 1924. Type: Mauritius: “Cum form. Praecentibus”, s.d., *J. B. G. M. Bory s.n.* (lectotype, designated here: GDC [G00324554]!). Syntype: Mauritius: s.l., s.d., *F. W. Sieber 18*2 GDC [G00324556]!.

Acalyphacommersonianavar.longifolia Müll.Arg., Prodr. 15(2): 850. 1866. Type: Mauritius: s.l., s.d., *Bouton s.n.* (lectotype, designated here: GDC [G00324553]!; isolectotypes: GDC [G00324552!, G00324551!], K [K000431101!], **syn. nov.**).

Acalyphacommersonianavar.parvifolia Baill. ex Müll.Arg., Prodr. 15(2): 850. 1866. Acalyphaintegrifoliavar.parvifolia (Baill. ex Müll.Arg.) Pax & K.Hoffm., Pflanzenr. 147,16(Heft 85): 106. 1924. Type: Mauritius: “Cum var. praecedentibus”, s.d., *F. W. Sieber 369* p.p. (holotype: GDC [G00324538]!, **syn. nov.**).

Acalyphacommersonianavar.obtusifolia Müll.Arg., Prodr. 15(2): 850. 1866. Acalyphacommersonianavar.obtusifoliaf.discolor Müll.Arg., Prodr. 15(2): 850. 1866. Type: Mauritius: “Cum praecedentibus”, s.d., *F. W. Sieber 181 pr. p.* (holotype: GDC [G00324558]!, **syn. nov.**).

Acalyphacommersonianavar.obtusifoliaf.unicolor Müll.Arg., Prodr. 15(2): 850. 1866. Type: Mauritius: s.l., s.d., *F. W. Sieber 178* (holotype: GDC [G00324539]!; isotypes P [P04779345!, P04780015!].

*Acalyphadiscolor* Bojer, Hortus Maurit. 286. 1837 *nom. nud*.

*Tragiamacrophylla* Wall., Numer. List n. 7796. 1847 *nom. nud*.

*Tragialobata* Wall., Numer. List n. 7796. 1847 *nom. nud*.

*Acalyphacommersoniana* Baill., Adansonia 1: 267. 1861 *nom. nud*.

Acalyphacommersonianavar.concolor Baill., Adansonia 1: 267. 1861 *nom. nud*.

Acalyphacommersonianavar.discolor Baill., Adansonia 1: 267. 186 *nom. nud*.

*Acalyphacommersonianavar.parvifolia* Baill., Adansonia 1: 267. 1861 *nom. nud*.

*Caturussessilis* Pet. Thou. ex Baill., Adansonia 1: 267. 1861 *nom. nud*.

*Tragiafruticosa* Commers. ex Baill., Adansonia 1: 267. 1861 *nom. nud*.

*Tragiaobtusata* Vahl. ex Baill., Adansonia 1: 267. 1861 *nom. nud*.

##### Distribution.

Mascarene Islands (Mauritius, Réunion).

##### References.

Bojer (1837: 286); Baillon (1858: 443) *A.colorata*; Müller Argoviensis (1866: 850); Baker (1877: 315) *A.colorata*; de Cordemoy (1895: 342) *A.colorata*; Palacký (1907: 24) *A.commersoniana*; Palacký (1907: 25); Pax and Hoffmann (1924: 105); Coode (1982: 69); Robertson (1989: 200); Govaerts et al. (2000: 69).

##### Notes.

Coode (1982) accepted three subspecies with six varieties within *Acalyphaintegrifolia*, but we find the varieties he placed within subsp. integrifolia to overlap too much to accept as distinct taxa. See *A.marginata* (Poir.) Spreng. for our treatment of what Coode treated within A.integrifoliasubsp.marginata (Poir.) Coode and subsp. panduriformis Coode.

#### 17. Acalypha lamiana

Taxon classificationPlantaeMalpighialesEuphorbiaceae

(Leandri) I.Montero & Cardiel. comb. nov.

urn:lsid:ipni.org:names:60476986-2

Acalypha reticulata var. urophylla f. lamiana Leandri, Notul. Syst. (Paris) 10: 263. 1942.

##### Type.

Madagascar, Prov. Mahajanga, Reserve de Marohogo, 28 Dec 1938, *H. J. Lam & A. D. J. Meeuse, 6127* (lectotype, designated here: P [P05604417]!; isolectotype: L [L0242109]!).

##### Syntypes.

Madagascar, Prov. Antananarivo, Tsarasaotra, Feb 1898, *H. Perrier de la Bâthie 457* P [P05604408!, P05604409!, P05604410!, P05604413!]; Madagascar, Prov. Fianarantsoa, Ankirihitra près du mont Tsilondroina, Mar 1902 , *H. Perrier de la Bâthie 9817* P [P05604403]!, *9817 bis* P [P05604404!, P05604405!]; Madagascar, Prov. Mahajanga, NW of Ankazobe,Vallée de l’Ikopa, 14 Mar 1930, *R. Decary 7535* P [P05604421]!; Massif de l’Ankarafantsika, 11 Jan 1938, *R. Decary 12876* P [P05604420]!; Bekodoka, 17 Sept 1930, *R. Decary 8109* P [P05604422]!; Region d’Antsalova, 1932-1933, *J. Leandri 998* P [P05604411]!; Tsingy du Bemaraha, 3-6 Oct 1932, *J. Leandri 176* P [P05604415]!; Dokolahy, Feb-Apr 1933, J. Leandri 602 P [P05604414]!; Madagascar, Prov. Toliara, Soahazo Forest, 100 m, 22 Oct 1932, *J. Leandri 414* P [P05604416]!.

##### Distribution.

Madagascar (Antananarivo, Fianarantsoa, Mahajanga and Toliara).

##### Notes.

*Acalyphalamiana* was treated by Leandri (1942) as a form of A.reticulatavar.urophylla (Boivin ex Baill.) Müll.Arg. (treated here as *A.urophylla*). After studying the numerous type collections, it seems clear to us that *A.lamiana* must be considered as a distinct species. *A.lamiana* differs from *A.urophylla* mainly by the leaves that are rounded at the base and reddish at the margins and its subentire, eglandular female bracts vs. leaves that are usually acute at the base and not reddish at the margins and dentate female bracts with small sessile glands at margins, in *A.urophylla*.

One of the mentioned syntypes, *Perrier de la Bâthie 9817*, was wrongly transcribed by Leandri (1942: 263) as “*8917*”.

#### 18. Acalypha lanceolata var. glandulosa

Taxon classificationPlantaeMalpighialesEuphorbiaceae

(Müll.Arg.) Radcl.-Sm., Kew Bull. 44(3): 444. 1989.

Acalypha crenata var. glandulosa Müll.Arg., Linnaea 34: 43. 1865.

##### Type.

Tanzania: Zanzibar, 1847-1852, *L. H. Boivin s.n.* (lectotype, designated here: P [P05511211]!; isolectotypes: P [P05511212!, P05511225!, P05510174!]).

##### Distribution.

*Acalyphalanceolata* Willd. is widely distributed in the Paleotropics; the var.glandulosa occurs in East Tropical Africa and South Tropical Africa. Madagascar (Antsiranana). Mascarene Islands (Réunion).

##### References.

Müller Argoviensis (1866: 872); Coode (1982: 69, 79); Govaerts et al. (2000: 71); Seebaluck et al. (2015: 152).

##### Notes.

Acalyphalanceolatavar.lanceolata occurs in Asia. The main diference between the African var.glandulosa and the Asian var.lanceolata is the presence or absence of stipitate glands. Additional studies are needed to clarify the taxonomic status of these taxa.

#### 19. Acalypha leonii

Taxon classificationPlantaeMalpighialesEuphorbiaceae

Baill., Bull. Mens. Soc. Linn. Paris ii. (1895) 1197.

##### Type.

Madagascar. Prov. Toamasina: Forêt d’Antsianaka, 14 Dec 1882, *L. Humblot 514* (lectotype, designated here: P [P00513056]!; isolectotypes: K [K000186528]!, P [P00513057!, P00513058!, P00513055!]).

Acalyphaleoniivar.perrierana Leandri, Notul. Syst. (Paris) 10: 271. 1942. Type: Madagascar. Prov. Mahajanga: bassin du Bemarivo, versant NE, 100 m, 1912, *H. Perrier de la Bâthie 9719* (lectotype, designated here: P [P00513061]!; isolectotypes: P [P00513059!, P00513060!], **syn. nov.**).

##### Distribution.

Madagascar (Antananarivo, Antsiranana, Toamasina, Toliara).

##### References.

Palacký (1907: 25); Pax and Hoffmann (1924: 112); Leandri (1942: 271); Leandri (1952); Govaerts et al. (2000: 72); Schatz (2001: 142).

#### 20. Acalypha lepidopagensis

Taxon classificationPlantaeMalpighialesEuphorbiaceae

Leandri, Notul. Syst. (Paris) 10: 280. 1942.

##### Type.

Madagascar. Prov. Antsiranana: Massif du Tsaratanana, 1000 m, Dec 1912, *H. Perrier de la Bâthie 9726* (lectotype, designated here: P [P00513062]!; isolectotype: P [P00513063]!).

##### Distribution.

Madagascar (Antsiranana).

##### References.

Leandri (1952); Govaerts et al. (2000: 72); Schatz (2001: 142).

#### 21. Acalypha leptomyura

Taxon classificationPlantaeMalpighialesEuphorbiaceae

Baill., Hist. Pl. Madag., Atlas (1891) t. 191.

##### Type.

Madagascar. Plate 191 in Baillon ibid. loc., holotype.

##### Distribution.

Madagascar (Antananarivo, Antsiranana, Fianarantsoa, Mahajanga, Toamasina and Toliara).

##### References.

Baillon (1892: 1004); Koehne (1892: 131); Palacký (1907: 25); Pax and Hoffmann (1924: 112); Leandri (1942: 271); Govaerts et al. (2000: 72).

#### 22. Acalypha linearifolia

Taxon classificationPlantaeMalpighialesEuphorbiaceae

Leandri, Notul. Syst. (Paris) 10: 275. 1942.

##### Type.

Madagascar. Prov. Toliara: Ambovombe, Kotoala, 21 Jan 1931, *R. Decary 8423* (lectotype, designated here: P [P00513090]!; isolectotypes: S [S07-14664]!, TAN [TAN000510]!, US [US01014148]!).

##### Syntypes.

Madagascar. Prov. Toliara: delta de la Linta, 17–24 Aug 1928, *H. Humbert & C. F. Swingle 5385* P [P00513086!, P00513087!, P00513088!, P00513089!]; US [US00096361]!.

##### Distribution.

Madagascar (Toamasina, Toliara).

##### References.

Govaerts et al. (2000: 72).

#### 23. Acalypha marginata

Taxon classificationPlantaeMalpighialesEuphorbiaceae

(Poir.) Spreng., Syst. Veg. 3: 879. 1826.

Tragia marginata Poir., Encycl. 7: 725. 1806. Acalyphaintegrifoliasubsp.marginata (Poir.) Coode, Kew Bull. 34: 42. 1979.

##### Type.

Mauritius: “Les Indes Orientales”, s.d. (holotype P-LAM [P00382145]!).

Acalyphaintegrifoliasubsp.panduriformis Coode, Kew Bull. 34: 42. 1979. Type: Réunion: Cliff between St Philippe and St Joseph near Basse vallée, ca. 100 m, 26 Feb 1975. *M. J. E. Coode & T. H. Cadet 4968* (holotype K [K000431108]!; isotype K [K000431107]!, **syn. nov.**).

Acalyphaintegrifoliasubsp.marginatavar.saltuum Coode, Kew Bull. 34: 43. 1979. Type: Mauritius: Macabé, 650 m, 15 Feb 1975, *M. J. E. Coode et al. 4874* (holotype: K [K000431106]!; isotypes P [P04779351]!, MAU [n.v.], **syn. nov.**).

Acalyphaintegrifoliasubsp.marginatavar.crateriana Coode, Kew Bull. 34: 44. 1979. Type: Mauritius: Tamarin Falls, 2 Mar1975, *D. Lorence 1138* (holotype: K [K000431104]!; isotype K [K000431105]!, MAU [n.v.], **syn. nov.**).

##### Distribution.

Mascarene Islands (Mauritius, Réunion).

##### References.

Bojer (1837: 286); Baillon (1858: 443); Baillon (1861: 267); Baker (1877: 315); de Cordemoy (1895: 343); Palacký (1907: 25); Pax and Hoffmann (1924: 106); Coode (1982: 73) A.integrifoliasubsp.panduriformis, A.integrifoliasubsp.marginata; Coode (1982: 74) A.integrifoliavar.saltuum, A.integrifoliavar.crateriana; Govaerts et al. (2000: 69) A.integrifoliasubsp.marginata; Seebaluck et al. (2015: 152).

##### Notes.

*Acalyphamarginata* was treated by Coode (1979) as A.integrifoliasubsp.marginata. We consider *A.marginata* to be a distinct species, differentiated mainly by its variegated leaf blades and vestigial female bracts that are not accrescent in fruit vs. non variegated leaf blades and conspicuous female bracts that are accrescent in fruit in *A.integrifolia*. We include as synonyms A.integrifoliasubsp.panduriformis, A.integrifoliavar.crateriana and A.integrifoliavar.saltuum because they have the same characters as *A.marginata*.

#### 24. Acalypha medibracteata

Taxon classificationPlantaeMalpighialesEuphorbiaceae

Radcl.–Sm. & Govaerts, Kew Bull. 52(2): 477. 1997.

Acalypha gagnepainii Leandri Notul. Syst. (Paris) 10: 274. 1942 *nom. illeg.* non A.gagnepainii Merr. (1938).

##### Type.

Madagascar. Prov. Toliara: Massif du Vohitsiombe (Fort-Dauphin), 31 Jul 1926, *R. Decary 4664* (lectotype, designated here: P [P00508417]!; isolectotype: S [S07-14667]!).

##### Syntypes.

Madagascar. Prov. Toliara: Vallée du Mandrare, s.d., *R. Decary 2620* P [P00508418]!; Madagascar. Prov. Toliara: Vallée de l’Ikonda, au N. d’Ambovombe, *R. Decary 8913*, P [P00887487]!; Madagascar. Prov. Toliara: Imangory, s.d., *R. Decary 8948* P [P00508416]!; Madagascar. Prov. Toliara: Beteny, (limite Nord-Est de l’Androy), 22 Nov 1931, *R. Decary 9355* P [P00508415]!, G [G00034242]!, GB [GB0047682]!.

Acalyphamedibracteatavar.calcicola (Leandri) Radcl.–Sm. & Govaerts, Kew Bull. 52(2): 477. 1997. Acalyphagagnepainiivar.calcicola Leandri Notul. Syst. (Paris) 10: 275. 1942. Type: Madagascar. Prov. Toliara: vallée moyenne du Mandrare près d’Anadabolava, Dec 1933, *H. Humbert 12422* (lectotype, designated here: P [P00508409]!; isolectotypes: P [P00508405!, P00508406!, P00508407!, P00508408!], **syn. nov.**).

Syntypes. Madagascar. Prov. Toliara: Fort-Dauphin, *J. Cloisel 18* P [P00508410]!, P [P00508411]!; Madagascar. Prov. Toliara: Basse vallée du Fiherenana, s.d., *H. Humbert 11573* P [P00508412!, P00508413!].

*Acalyphamenabeana* Leandri Notul. Syst. (Paris) 10: 275. 1942 *nom. nud.* as *A.gagnepainii*.

*Acalyphaaspretorum* Leandri Notul. Syst. (Paris) 10: 275. 1942 *nom. nud*. as *A.gagnepainii*.

##### Distribution.

Madagascar (Antananarivo, Toamasina, Toliara).

##### References.

Govaerts et al. (2000: 75, 100).

#### 25. Acalypha menavody

Taxon classificationPlantaeMalpighialesEuphorbiaceae

(Leandri) I.Montero & Cardiel comb. nov.

urn:lsid:ipni.org:names:60476988-2

Acalypha spiciflora var. menavody Leandri, Notul. Syst. (Paris) 10: 270. 1942.

##### Type.

Madagascar. Prov. Antsiranana: Collines et plateaux calcaires de l’Analamera, Jan 1938, *H. Humbert 19149* (lectotype, designated here: P [P00536737]!; isolectotypes: P [P00536736!, P00536738!]).

##### Distribution.

Madagascar (Antsiranana).

##### Notes.

*Acalyphaspiciflora* has been excluded because it does not belong to *Acalypha* (see notes under this name). We only recognise A.spicifloravar.menavody as *A.menavody*.

#### 26. Acalypha paxii

Taxon classificationPlantaeMalpighialesEuphorbiaceae

Aug.D.C., Bull. Herb. Boissier, sér. 2, 1: 567. 1901.

Acalypha urophylla Pax, Bot. Jahrb. Syst. 19: 96. 1894. *nom. illeg*. non A.urophylla Boivin ex Baill. (1861). Acalyphareticulatavar.urophylloides Pax & K.Hoffm., Pflanzenr. 147, 16 (Heft 85): 105. 1924. Acalyphafiliformisvar.urophylloides (Pax & K.Hoffm.) Govaerts, World Checkl. Bibliogr. Euphorbiaceae 63. 2000.

##### Type.

Madagascar. Prov. Antsiranana: Nossibé, nordwest, Feb 1880, *J. M. Hildebrandt 3356* (holotype B or WRSL, presumably destroyed; lectotype, designated here: BREM [BREM0001784]!; isolectotypes: JE [JE00004294!, JE00004293!], K [K000186531]!, M [M0110600]!, P [P00536741!, P00536742!, P00536743!]).

*Acalyphapervilleana* Baill., Adansonia 1: 273. 1861. Acalyphareticulatavar.pervilleana (Baill.) Müll.Arg., Linnaea 34: 32. 1865. Acalyphafiliformisvar.pervilleana (Baill.) Govaerts, World Checkl. Bibliogr. Euphorbiaceae 62. 2000. Type. Madagascar. Prov. Antsiranana: Nossibé, 1840, *M. Richard 384* (lectotype, designated here: P [P00536745]!; isolectotype: P [P05604474]!, **syn. nov.**).

Syntypes. Madagascar, Prov. Antsiranana: Nossibé, *M. Pervillé 368* P [P00536746!, P00536747!].

*Acalyphahumblotiana* Baill., Hist. Pl. Madag., Atlas (1891) t. 1891. Acalyphareticulatavar.urophyllaf.humblotiana (Baill.) Leandri, Notul. Syst. (Paris) 10: 262. 1942. Type: Comoros: Grande comore, 14 Nov 1885, *L. Humblot 1461* (lectotype, designated here: P [P00196295]!; isolectotypes: P [P02712292!, P00196296!], **syn. nov**.).

*Acalyphameiodonta* Baill., Bull. Mens. Soc. Linn. Paris 2: 1197. 1895. Acalyphareticulatavar.meiodonta (Baill.) Leandri. Notul. Syst. (Paris) 10: 267. 1942. Type: Madagascar: Centr. Madag., Dec. 1883, *R. Baron 2826* (lectotype, designated here: P [P05604378]!; isolectotype: K [K000186508]!, **syn. nov.**).

Syntype: Madagascar: “Centr. Madag.”, s.d., *R. Baron 6581* K [n.v.].

Acalyphareticulatavar.meiodontaf.andronea Leandri, Notul. Syst. (Paris) 10: 268. 1942. Type: Madagascar, Prov. Mahajanga, Bemarivo (Boïna), Dec 1906, *H. Perrier de la Bâthie 9561* (lectotype, designated here: P [P00513143]!; isolectotype: P [P00513144]!, **syn. nov.**).

Former syntypes: Madagascar, Prov. Mahajanga, bord du massif du Manongarivo, versant du Sambirano, 1909, *H. Perrier de la Bâthie 9934* P [P00513149]!; Massif du Manongarivo, versant du Sambirano, Sep 1909, *H. Perrier de la Bâthie 9939* P [P00513150]!; haut Bemarivo (Andranofosy), Boïna, Jan 1907, *H. Perrier de la Bâthie 9635* P [P00513145]!; Massif du Manongarivo, Sambirano, Apr 1909, *H. Perrier de la Bâthie 9928* P [P00513142]!; Manongarivo (Ambongo), Oct 1904, *H. Perrier de la Bâthie 1677*, P [P00513146!, P00513147!].

##### Distribution.

Madagascar (Antsiranana, Mahajanga, Toamasina). Comoros Archipelago (Grande Comore, Anjouan, Mohéli and Mayotte).

##### References.

Müller Argoviensis (1866: 852) as A.reticulatavar.pervilleana; Müller Argoviensis (1882: 26) as A.reticulatavar.urophylla; Müller Argoviensis (1866: 852) as A.reticulatavar.pervilleana; Baillon (1892: 1004) as *A.pervilleana*; Baillon (1895b: 1197) as *A.humblotiana*; Palacký (1907: 25, 26); Pax and Hoffmann (1924: 105, 112) as Acalyphareticulatavar.urophylloides and A.reticulatavar.pervilleana; Leandri (1942: 258, 260) as A.reticulatavar.pervilleana; Govaerts et al. (2000: 62, 63, 75, 104 105, 107).

##### Notes.

*Acalyphapaxii* was proposed by August De Candolle as a replacement name for the illegitimate *A.urophylla* Pax. Leandri (1942) treated *A.paxii* as a synonym of A.reticulatavar.pervilleana. We consider that *A.paxii* is a well-differentiated species.

*Acalyphapaxii, A.pervilleana* and *A.meiodonta* have been considered synonyms of *A.reticulata*, which we consider to be a synonym of *A.filiformis*. *A.paxii* can be differentiated from *A.filiformis* by its sessile, dentate female bracts with a prominent central tooth vs. pedicellate, crenate to subentire female bracts in *A.filiformis* (see notes in *A.filiformis*).

*Acalyphapaxii* can be distinguished from *A.urophylla*, which has also been placed within *A.reticulata*, mainly by its leaves with usually caudate apices, its dentate female bracts with a prominent central tooth and eglandular margins vs. leaves with usually acuminate apices, dentate female bracts without a prominent central tooth and with small sessile glands at the margins. See notes under *A.gracilipes* Baill. for the differences between *A.paxii* and that species.

*Acalyphahumblotiana* was first illustrated, without description, in Baillon`s (1891)*Histoire Naturelle des Plantes*, but this illustration is not consistent with Baillon`s (1895b) later description nor with the specimen on which the description presumably is based (*L. Humblot 1461*).

Although *Richard 384* [P00536745]! and *385* [P04779454]! have labels giving the locality as “Bourbon”, now Réunion, these are not Richard’s original labels and the species is otherwise unknown from the Mascarene Islands. We do not have evidence that it occurs there.

#### 27. Acalypha perrieri

Taxon classificationPlantaeMalpighialesEuphorbiaceae

Leandri, Notul. Syst. (Paris) 10: 273. 1942.

##### Type.

Madagascar. Prov. Mahajanga: Belambo, près de Maevatanana, Aug 1901, *H. Perrier de la Bâthie 981* (lectotype, designated here: P [P00513095]!).

##### Syntypes.

Madagascar. Prov. Mahajanga: Menabé, Tsiampihy, *J. Leandri 275* P [P00513092]!, P [P00513093]!, TAN [TAN000511]!; Madagascar. Prov. Mahajanga: ibid. loc., s.d., *J. Leandri 294*, P [P00513091]!; Madagascar. Prov. Mahajanga: Forêt de Tsimembo, s.d., *J. Leandri* 420, P [P00513094]!.

##### Distribution.

Madagascar (Antsiranana, Mahajanga, Toliara).

##### References.

Govaerts et al. (2000: 81).

#### 28. Acalypha poiretii

Taxon classificationPlantaeMalpighialesEuphorbiaceae

Spreng., Syst. Veg. 3: 879. 1826.

Ricinocarpus poiretii (Spreng.) Kuntze, Gen. Pl. 2: 618. 1891.

##### Type.

‘‘Amer. trop.’’ s. loc., s.d., *Anonymous s.n.* (holotype: P-LAM [P00382110]!).

##### Distribution.

Introduced in Tropical Africa and the WIOR region. Mascarene Islands (Mauritius, Réunion, Rodríguez).

##### References.

Baker (1877: 315); de Cordemoy (1895: 312); Coode (1982: 79).

##### Notes.

Herb native to the Americas. It has been reported from continental Africa (Cardiel and Montero Muñoz 2018) and from the Mascarene Islands (Baker 1877; de Cordemoy 1895; Coode 1982). We found specimens from the Mascarene Islands (Mauritius, Réunion and Rodríguez).

#### 29. Acalypha radula

Taxon classificationPlantaeMalpighialesEuphorbiaceae

Baker, J. Linn. Soc., Bot. 20: 254. 1883.

##### Type.

Madagascar: “Central Madagascar”, 1882, *R. Baron 1818* (holotype: K [K000186509]!; isotypes: P [P00513119!, P00513120!]).

*Acalyphahildebrandtii* Baill., Bull. Mens. Soc. Linn. Paris 2: 1005, 1180. 1892. Type: Madagascar. Prov. Fianarantsoa: “Betsileo, Nandahizana,”. *J. M. Hildebrandt 3900* (holotype: P [P00513121]; isotypes: BREM [BREM0001783]!, G [G00190630!, G00074184!], JE [JE00000289!, JE00000288!], K [K000186507]!, M [M0110604]!, P [P00513122]!).

*Acalyphaandringitrensis* Leandri, Notul. Syst. (Paris) 10: 277. 1942. Type. Madagascar. Prov. Fianarantsoa: Massif of Andringitra, Apr 1921, *H. Perrier de la Bâthie 13640* (lectotype, designated here: P [P00508596]!; isolectotypes: P [P00508594!, P00508595]!, **syn. nov**.).

Syntypes. Madagascar, ibid. loc., 1924, *H. Humbert 3709* P [P00224706]!, P [P00508591]!, P [P00508592]!, P [P00508593]!; Madagascar, ibid. loc., Apr 1921, *H. Perrier de la Bâthie 9671* P [P00508589!, P00508590!].

*Acalyphasalviifolia* Baill., Étude Euphorb. 443. 1858 *nom. nud*.

*Tragiasalviaefolia* Boj. ex Baill., Étude Euphorb. 443. 1858 *nom. nud*.

##### Distribution.

Madagascar (Antananarivo, Fianarantsoa, Toamasina and Toliara).

##### References.

Baillon (1861: 268) as *A.salviifolia*; Müller Argoviensis (1866: 889) *A.salviifolia*; Baillon (1891: 193); Baillon (1892: 1004) *A.salviifolia*; Baillon (1895a: 1180) *A.hildebrandtii*; Palacký (1907: 25) *A.hildebrandtii*; Palacký (1907: 26); Palacký (1907: 26) *A.salviifolia*; Pax and Hoffmann (1924: 102) as *A.salviifolia*; Pax and Hoffmann (1924: 156) *A.hildebrandtii*; Leandri (1942: 278); Leandri (1952); Jenkins (1987:347); Jenkins (1990: 408, 433); Goodman (1996: 61) *A.andringitrensis*; Govaerts et al. (2000: 49) *A.andringitrensis*; Govaerts et al. (2000: 67) *A.hildebrandtii*; Govaerts et al. (2000: 85, 86, 105) *A.salviifolia*; Schatz (2001: 142); Seebaluck et al. (2015: 149, 153).

##### Notes.

Although Leandri (1942) distinguished *Acalyphaandringitrensis* and *A.radula* based on leaf shape and bract incision, more recent collections show continuous variation between the extremes recognised by Leandri, and DNA sequences do not differentiate these forms (G. A. Levin, pers. obs.).

#### 30. Acalypha richardiana

Taxon classificationPlantaeMalpighialesEuphorbiaceae

Baill., Adansonia 1: 268. 1861.

##### Type.

Comoros: Mohéli, “Ile Mohilla”, s.d., *M. Richard 287* (lectotype, designated here: P [P04779566]!; isolectotypes: P [P04779562!, P04779564!, P04779565!]).

##### Syntypes.

Madagascar: s.l., s.d., *M. Richard 544* [P04779563]!; Mayotte, s.l., s.d., *L. H. Boivin 3373* GDC [G00324505]!, P [P00196299!, P00196300!], W [W-Rchb. 1889-0166704]!.

*Acalyphaovalifolia* Baill., Adansonia 1: 269. 1861. Acalyphareticulatavar.ovalifolia (Baill.) Müll.Arg., Linnaea 34: 32. 1865. Acalyphafiliformisvar.ovalifolia (Baill.) Govaerts, World Checkl. Bibliogr. Euphorbiaceae 62. 2000. Type: Mayotte: Nov 1848. *L. H. Boivin 3372* (holotype: P [P00196298]!).

##### Distribution.

Comoros Archipelago (Anjouan, Mohéli, Mayotte).

##### References.

Müller Argoviensis (1866: 852 as A.reticulatavar.ovalifolia, 855); Baillon (1892: 1004); Palacký (1907: 25 as *A.ovalifolia*, 26); Voeltzkow (1917: 447); Pax and Hoffmann (1924: 127); Leandri (1942: 272); Govaerts et al. (2000: 62, 85, 105).

##### Notes.

The specimens, indicated as isolectotypes, do not have Richard’s original label. They instead have labels with Baillon’s handwriting giving the location as “Madagascar”. We believe that the correct location is the one indicated on the selected lectotype, “Ile Mohilla”, whose current name is Mohéli, in the Comoros Archipelago. A search of specimens at P showed that Richard’s collections numbered *284–286*, *288*, *290*, *291* and *293* are also from Mohéli (*289* has no locality and *292* is not listed). *Richard 544* has an apparently original label showing the locality only as “Madagascar.” Collections at P with nearby numbers are labelled as being from either “Nord de Madagascar” or ”Ile Nos-bé” (now Nossi-bé), so this collection could be from northern Madagascar, although we have seen no other specimens from outside the Comoros Archipelago.

#### 31. Acalypha rottleroides

Taxon classificationPlantaeMalpighialesEuphorbiaceae

Baill., Adansonia 1: 270. 1861.

##### Type.

Madagascar. Prov. Antsiranana: Nossibé, 1837, *M. Richard 215* (holotype P [P00536728]!).

*Acalyphapolynema* Baill., Hist. Pl. Madag., Atlas (1891) t. 187. Type: Madagascar: Plate 187 in Baillon ibid. loc., holotype.

*Acalyphajuliflora* Pax, Bot. Jahrb. Syst. 19: 95. 1894. Type: Madagascar. Prov. Antsiranana: Nossibé, “Urwald von Loko-bé”, Dec 1879, *J. M. Hildebrandt 3279* (holotype: W [W1889-0089773]!; isotypes: JE [JE00004291]!, K [K000186527]!, P [P00536729!, P00536730!, P00536731!]).

##### Distribution.

Madagascar (Antsiranana).

##### References.

Müller Argoviensis (1866: 854); Baillon (1891: 182); Baillon (1895b: 1197); Baillon (1895b: 1197) *A.polynema*; Palacký (1907: 25) *A.juliflora*; Palacký (1907: 26) *A.polynema*; Palacký (1907: 26); Nitschke (1923: 280) *A.juliflora*; Pax and Hoffmann (1924: 127); Leandri (1942: 277); Govaerts et al. (2000: 70, 105) *A.juliflora*; Govaerts et al. (2000: 86).

#### 32. Acalypha spachiana

Taxon classificationPlantaeMalpighialesEuphorbiaceae

Baill., Adansonia 1: 272. 1861.

Acalypha spachiana var. latifolia Baill., Adansonia 1: 272. 1861.

##### Type.

Madagascar. Prov. Antsiranana: Baies de Rigny et de Diego-Suarès, 1848, *L. H. Boivin 2654* (lectotype, designated here: P [P00536733]!; isolectotype: G [G00034251]!, GDC [G00324359]!, P [P00536734]!).

Acalyphaspachianavar.acutifolia Baill., Adansonia 1: 272. 1861. Type: Madagascar. Prov. Antananarivo: Antananarivo, 12 Feb 1840, *J. P. Goudot s.n.* (holotype: G [G00383582]!, **syn. nov**.).

Acalyphaspachianavar.minor Baill., Adansonia 1: 272. 1861. Type: Madagascar: s.l., s.d., *M. Bojer s.n.* (holotype: P [P00536735]!; isotypes: TUB [TUB002081!, TUB002082]!, **syn. nov**.).

*Acalyphabuchenavii* Müll.Arg., Abh. Naturwiss. Verein Bremen 7: 27. 1880. Type: Madagascar. Prov. Antananarivo: Antananarivo, 18 Dec. 1877, *D.C. Rutenberg s.n*. (lectotype (probably holotype), designated here: BRNU [BRNU347926]!).

*Acalyphasquarrosa* Pax, Bot. Jahrb. Syst. 19: 97. 1894. Type. Madagascar. Prov. Antananarivo: Antsirabe, “Sírabé”, Aug 1880, *J. M. Hildebrandt 3560* (holotype B or WRSL, presumably destroyed; lectotype, designated here: JE [JE00004308]!; isolectotype: JE [JE00004309]!, K [K000186510]!, P [P00536732]!).

*Tragiasaxatilis* Bojer ex Pax & K.Hoffm., Pflanzenr. 147, 16 (Heft 85): 33. 1924 *nom. nud*.

##### Distribution.

Madagascar (Antananarivo, Antsiranana, Mahajanga, Toamasina, Toliara).

##### References.

Müller Argoviensis (1866: 827); Müller Argoviensis (1882: 27) *A.buchenavii*; Baillon (1892: 1003); Baillon (1895b: 1199) *A.buchenavii*; Palacký (1907: 26); Pax and Hoffmann (1924: 33); Pax and Hoffmann (1924: 33) *A.squarrosa*; Leandri (1942: 255, 257); Jenkins (1987: 347); Jenkins (1990: 408, 433); Govaerts et al. (2000: 89, 96); Govaerts et al. (2000: 89) *A.squarrosa*; Seebaluck et al. (2015: 153).

##### Notes.

The holotype of *Acalyphabuchenavii* should be at BREM but Rutenberg’s specimens arrived at BRNU after World War II. Originally in the Überseemuseum in Bremen (BREM), they were transferred to northern Moravia (Czechia was at that time a Protectorate of Nazi Germany) to save them from potential destruction by bombardment. After the collapse of the Nazi regime, these collections were confiscated as “German property” and sent to BRNU.

#### 33. Acalypha urophylla

Taxon classificationPlantaeMalpighialesEuphorbiaceae

Boivin ex Baill., Adansonia 1: 273. 1861.

Acalypha reticulata var. urophylla (Boivin ex Baill.) Müll.Arg. Linnaea 34: 32. 1865.
Acalypha filiformis var. urophylla (Boivin ex Baill.) Govaerts, World Checkl. Bibliogr. Euphorbiaceae 63. 2000.
**Type.** Madagascar. Prov. Antsiranana: Nossibé, plateau de Hell-Ville, Jun 1847, *L. H. Boivin 2178* (lectotype, designated here: P [P00536752]!; isolectotypes: G [G00034246]!, GDC [G00324519]!, P [P00536751!, P00536753!]).
**Syntypes.** Madagascar: s.l., s.d., *L. M. A. Du Petit-Thouars s.n.* P [P00536748]!; Madagascar. Prov. Antsiranana: Nossibé, Dec 1840, *A. Pervillé 364* P [P00536749]!; Madagascar. Prov. Antsiranana: Nossibé, *M. Richard 385* P [P00536750]!.
Acalypha goudotiana Baill., Adansonia 1: 268. 1861. Acalyphareticulatavar.goudotiana (Baill.) Müll.Arg., Linnaea 34: 32. 1865. Acalyphafiliformisvar.goudotiana (Baill.) Govaerts, World Checkl. Bibliogr. Euphorbiaceae 62. 2000. Type: Madagascar: s.l., 1830, *J. P. Goudot s.n.* (holotype: G!; isotype: P [P00536727]! fragment), **syn. nov.**
Acalypha reticulata var. urophylla f. meeusei Leandri, Notul. Syst. (Paris) 10: 264. 1942. Type: Madagascar, Prov. Fianarantsoa, base Est du Pic d’Ivohibe, 19 Sep 1926, R. Decary 5352 P [P00224690]!; Prov. Toamasina, Analamazaotra forest, 1912, *H. Perrier de la Bâthier 9741* (lectotype, designated here: P [P05604377]!, **syn. nov.**).
Syntypes. Madagascar, Prov. Toamasina, Soanierana, Antasibé, 9 Dec 1938, *H. J. Lam & A. D. J. Meeuse 5814* WAG [WAG0133229]!, L [L0242110]!; Moramanga, 900 m, 11 Nov 1938, H. J. Lam & A. D. J. Meeuse 5363 L [L0242105]!; Analamazaotra, 1000 m, 10 Nov. 1938, *H. J. Lam & A. D. J. Meeuse 5290* L [L0242106]!.
Acalypha reticulata var. urophylla f. longa Leandri, Notul. Syst. (Paris) 10: 265. 1942. Type: Madagascar, Prov. Toliara, bassin de la Manampanihy, Col de Fitana, 700 m, 15 Oct 1928, H. Humbert 6015 (lectotype, designated here: P [P05604383]!, **syn. nov.**).
Syntypes. Madagascar, Prov. Toliara, Col d’Ivolo (District de Fort-Dauphin), 500 m, 5 Sept 1932, *R. Decary 10558* P [P05604384]!; Fort-Dauphin, col de Tsitongabarika, 600 m, 9 Sept 1932, *R. Decary 10595* P [P05604399]!.
Acalypha reticulata var. urophylla f. vohitrae Leandri, Notul. Syst. (Paris) 10: 265. 1942. Type: Madagascar, Prov. Toamasina, Andevorante, rive droite de la Vohitra près de Lohariandava, 200-250 m, 10 Oct 1912, *R. Viguier & H. Humbert 661* (lectotype, designated here: P [P05604437]!; isolectotypes: P [P05604440!, P05604441!], **syn. nov.**).
Acalypha reticulata var. urophylla f. glabrescens Leandri, Notul. Syst. (Paris) 10: 266. 1942. Type: Madagascar, Prov. Antsiranana, Massif du Tsaratanana, 2200 m, H. Perrier de la Bâthie 16180 (lectotype, designated here: P [P05604386]! **syn. nov.**).
Syntypes. Madagascar, Prov. Antsiranana, Sambirano, Nov-Dec 1937, *H. Humbert 18659* P [P05604397!, P05604400!]; Massif du Tsaratanana, Sep 1912, *H. Perrier de la Bâthie 18614* P [P05604382]!; Massif du Tsaratanana, 1600 m, H. Perrier de la Bâthie 15371 P [P05604385]!; Madagascar, Prov. Fianarantsoa, Ranohira, Isalo, 30 Jul 1928, *H. Humbert 5014* P [P05604401!, P05604402!]; Madagascar, Prov. Mahajanga, Beritsoka, *H. Perrier de la Bâthie 413* P [P05604392!, P05604390!]; Beritsoka, Dec 1897, *H. Perrier de la Bâthie 422* P [P05604387!, P05604388!, P05604391!]; Beritsoka, H. Perrier de la Bâthie 9822 P [P05604389]!; Madagascar, s. l., *R. Baron 5987* P [P05604407]!; s. l., *L. Humblot 335* P [P05604393!, P05604394!, P05604395!, P05604396!].
Acalypha reticulata var. urophylla f. typique Leandri, Notul. Syst. (Paris) 10: 262. 1942 *nom. inval*.

##### Distribution.

Madagascar (Antananarivo, Antsiranana, Fianarantsoa, Mahajanga, Toamasina and Toliara). Comoros Archipelago (Grande Comore, Anjouan, Mohéli and Mayotte).

##### References.

Müller Argoviensis (1866: 260, 852) as A.reticulatavar.goudotiana and A.reticulatavar.urophylla; Baron (1889: 262); Baillon (1891: 189); Baillon (1892: 1004); De Candolle (1901: 567); Palacký (1907: 25 as *A.goudotiana*, 26); Pax and Hoffmann (1924: 105, 260) as as A.reticulatavar.goudotiana and A.reticulatavar.urophylla; Leandri (1942: 258, 260, 262) as A.reticulatavar.goudotiana and A.reticulatavar.urophylla; Leandri (1942: 281) as *A.emirnensis*; Leandri (1948: 186) as A.reticulatavar.goudotiana; Govaerts et al. (2000: 62, 63, 100, 105, 108).

##### Notes.

*Acalyphaurophylla* has been considered to be a variety of *A.reticulata*, but *A.urophylla* can be distinguished by its sessile female bracts with dentate margins vs. pedicellate female bracts with crenate to subentire margins in *A.filiformis*. (see notes in *A.filiformis*). See notes under *A.gracilipes*, *A.lamiana* and *A.paxii* for the differences between those species and *A.urophylla*.

#### 34. Acalypha vulneraria

Taxon classificationPlantaeMalpighialesEuphorbiaceae

Baill., Bull. Mens. Soc. Linn. Paris 2: 1180-1181. 1895.

##### Type.

Madagascar. Prov. Toliara: Fort-Dauphin, s.d., *G. F. Scott-Elliot 3010* (lectotype, designated here: P [P00536740]!; isolectotypes: K [K000186511]!, P [P00536739]!).

##### Syntypes.

Madagascar. Prov. Toliara: Fort-Dauphin, *M. Cloisel 51* (wrongly transcribed as “*Cloisel 50*” in the protologue) P [P04779526]!.

##### Distribution.

Madagascar (Fianarantsoa, Mahajanga, Toliara).

##### References.

Palacký (1907: 26); Nitschke (1923: 281); Pax and Hoffmann (1924: 128); Leandri (1935: 46); Leandri (1942: 279); Govaerts et al. (2000: 93).

#### 35. Acalypha wilkesiana

Taxon classificationPlantaeMalpighialesEuphorbiaceae

Müll.Arg., Prodr. 15(2): 817. 1866.

Ricinocarpus wilkesianus (Müll.Arg.) Kuntze, Revis. Gen. Pl. 2: 618. 1891. AcalyphaamentaceaRoxb.subsp.wilkesiana (Müll.Arg.) Fosberg, Smithsonian Contr. Bot. 45: 10. 1980.

##### Type.

Fiji, s.d., (U.S. Expl. Exped. Under. Capt. Wilkes), *B. C. Seeman s.n.* (holotype: G-DC [G00324022]!; isotypes: GH [00045512]!, K [K000959008]!, US [00096423!, 00096424!]).

##### Distribution.

Tropical Africa. Madagascar (Antananarivo).

##### References.

Robertson (1989: 200).

##### Notes.

Shrub native to Fiji (Melanesia), used as an ornamental plant throughout the tropics. It has been reported from Seychelles (Robertson 1989). We found some specimens from Madagascar

### Doubtful or excluded species

*Acalyphaneptunica* Müll.Arg., Abh. Naturwiss. Vereins Bremen 7: 26. 1880. Type. Tanzania, Zanzibar: Kidosi, Oct 1873, *J. M. Hildebrandt 1146*. (lectotype, designated by Cardiel and Montero Muñoz (2018): K [K000431078]!; isolectotype: G [G00007675]!).

This species occurs in West Tropical Africa, West Central Tropical Africa, Northeast Tropical Africa and East Tropical Africa. There are only two references of *Acalyphaneptunica* from Madagascar. They appear in Baillon (1895b) and in the checklist of Palacký (1907). We have not yet confirmed the presence of this species in the study area.

*Acalyphaspiciflora* Burm.f., Fl. Ind. 203, pl. 61-2. 1768. *Claoxylonspiciflorum* (Burm.f.) A.Juss., Euphorb. Gen. 43. 1824. *Cleidionspiciflorum* (Burm.f.) Merr., Interpr. Herb. Amboin. 322. 1917.

*Acalyphaspiciflora* Burm.f. was described and illustrated in Burman´s *Flora Indica*, but the plate and the description are very imprecise. Subsequently, Poiret (1804) cited under this name a specimen in the Lamarck herbarium, P00382113, from Réunion. That specimen is morphologically close to Burman´s plate, but it is not an *Acalypha* species. Later, Jussieu combined *A.spiciflora* under the genus *Claoxylon*, as *Cla.spiciflorum*. Merrill combined it under *Cleidion* as *Cle.spiciflorum*, the name that is accepted today. Müller Argoviensis (1866) wrongly placed *A.spiciflora* as a synonym of *A.acuminata* Vahl ex Baill. Pax and Hoffmann (1924) and Leandri (1942) followed the treatment of Müller Argoviensis, but, applying the rule of priority, chose *A.spiciflora* as the accepted name. This is the origin of the confusion in the use of this name.

*Acalyphavenosa* Poir., Encycl. 6: 204. 1804.

The type specimen of *Acalyphavenosa* (*Commerson s.n.*, from Madagascar) corresponds to *Leptonemavenosum* (Poir.) A.Juss. (Phyllanthaceae), as was pointed out by Steudel (1841: 31).

## Supplementary Material

XML Treatment for Acalypha bailloniana

XML Treatment for Acalypha boinensis

XML Treatment for Acalypha burmanii

XML Treatment for Acalypha chibomboa

XML Treatment for Acalypha claoxyloides

XML Treatment for Acalypha decaryana

XML Treatment for Acalypha diminuta

XML Treatment for Acalypha emirnensis

XML Treatment for Acalypha fasciculata

XML Treatment for Acalypha filiformis

XML Treatment for Acalypha fimbriata

XML Treatment for Acalypha gracilipes

XML Treatment for Acalypha hispida

XML Treatment for Acalypha humbertii

XML Treatment for Acalypha indica

XML Treatment for Acalypha integrifolia

XML Treatment for Acalypha lamiana

XML Treatment for Acalypha lanceolata var. glandulosa

XML Treatment for Acalypha leonii

XML Treatment for Acalypha lepidopagensis

XML Treatment for Acalypha leptomyura

XML Treatment for Acalypha linearifolia

XML Treatment for Acalypha marginata

XML Treatment for Acalypha medibracteata

XML Treatment for Acalypha menavody

XML Treatment for Acalypha paxii

XML Treatment for Acalypha perrieri

XML Treatment for Acalypha poiretii

XML Treatment for Acalypha radula

XML Treatment for Acalypha richardiana

XML Treatment for Acalypha rottleroides

XML Treatment for Acalypha spachiana

XML Treatment for Acalypha urophylla

XML Treatment for Acalypha vulneraria

XML Treatment for Acalypha wilkesiana
